# Effects of Boric Acid on Oxidative Stress Parameters, Growth Performance and Blood Parameters of Rainbow Trout (*Oncorhynchus Mykiss*)

**DOI:** 10.1007/s12011-024-04276-4

**Published:** 2024-06-24

**Authors:** Mustafa Öz

**Affiliations:** Department of Fisheries and Diseases, Faculty of Veterinary Medicine, Aksaray University, Aksaray, Turkey

**Keywords:** Boric acid, Rainbow trout, Growth parameters, Hematology, Blood biochemistry

## Abstract

Rainbow trout (*Oncorhynchus mykiss*) with a starting weight of 397.28 ± 3.21 g were fed different ratios (G1-0.00%, G2-0.010%, G3-0.025%, and G4-0.050%) of boric acid-supplemented feed for 140 days. The effects of dietary boric acid on oxidative stress parameters, growth performance, haematology and some biochemical parameters were investigated after the feeding period. The addition of boric acid to trout feed positively affected growth performance; the final weights of the groups were 928.15 ± 5.73 g, 955.87 ± 8.67 g, 994.24994,75 ± 7.46 g, and 976.80976,80 ± 6.26 g for the control group and the three experimental groups, respectively. The lowest feed conversation ratio (FCR) was 1.19 (G3) whereas the highest was 1.42 (G1). The lowest protein efficiency ratio was 1.63 (G1), while the highest was 1.95 (G3). In this study, it was observed that boric acid added to the feed changed muscle and blood oxidative stress parameters in rainbow trout, increased the growth performance of rainbow trout, and affected blood and biochemistry values.

## Introduction

The aquaculture industry is one of the fastest growing food sectors worldwide and plays an important role in ensuring global food security. While aquaculture hunting has decreased in recent years, aquaculture has been increasing very rapidly. Currently, more than half of the fish consumed for human food is produced by the aquaculture sector, and this level of production is predicted to increase further [1]. Therefore, strategies are required to ensure the sustainable growth of fish farming, which is crucial both environmentally and economically [2].

Rainbow trout ranks 13th among the world's most commonly farmed fish species, and the production of rainbow trout climbed from 340 thousand tonnes in 2000 up to approximately 740 thousand tonnes in 2020 [3]. To achieve sustainability in the farming of carnivorous species like rainbow trout, it is important to reduce or eliminate the proportions of fishmeal and fish oil in their feed. Substituting vegetable protein sources for fish meal, a fundamental ingredient in the feed regimens of carnivorous animals, has a negative impact on growth performance. Further investigation is required in this field to effectively and efficiently include additional vegetable protein and oil sources into fish meals in a sustainable and productive manner.

Many studies have been carried out to increase the growth performance of fish, protect fish health, and improve meat quality [4–13].

Boron is a vital element at low levels, but it can be harmful to plants and animals when present at higher amounts [14]. Although it is not well defined how boron functions in animals, it is necessary for nutrition and physiological processes. In addition, boron plays a role in immune response, bone growth, and mineral and endocrine metabolisms [15–17]. It is required for embryonic development, larval stages, and growth of zebrafish (*Danio rerio*) and rainbow trout (*Oncorhyncus mykiss*) [18–20]. It has been reported that water-borne boron affects the serum biochemical parameters of Nile tilapia adversely but does not change hematological parameters. In addition, it has been reported that high levels of boron in the aquatic ecosystem harm fish health [21]. It is known that blood parameters and serum biochemistry parameters are affected adversely as a result of long-term and high-dose use of boron in rainbow trout feeds [22], and it also causes damage to the gills and liver [23].

There are also studies which indicate that boric acid positively affects growth performance [19] and changes muscle nutrient content [12] and the fatty acid profile [13] of rainbow trout when used in trace amounts. In addition, it is stated that the results will change when boric acid is applied in different size groups and over different periods of time [12]. In contrast to previous studies, the current investigation utilised smaller amounts of boron to examine its impact on muscle and blood oxidative stress markers. Additionally, this study is the first to test the effects of boric acid on large-sized rainbow trout.

## Materials and Methods

### Preparation of the Fish and Diet

The present study obtained approval from the Animal Experiments Local Committee in Adana, Turkey (No. 9–1/2021). Before the feeding trial, 108 rainbow trouts with an initial weight of 397.28 ± 3.21 g underwent a 14-day acclimation period, during which they were fed a commercial diet comprising 43% crude protein, 24% crude lipid, 3.9% crude cellulose, and 9% crude ash (Skretting Stavanger, Norway). This commercial diet was also used as the control diet for the feeding trial. Four different diets with varying levels of boric acid (G1: 0.00%, G2: 0.010%, G3: 0.025%, G4: 0.050% of boric acid) were prepared by incorporating boric acid into the feed. The selection of boric acid levels was based on previous studies [19, 24]. The fish were randomly distributed across 12 net cages (each measuring 1 m^3^ with a mesh size of 10 mm), with a stocking density of 9 fish per cage. Following the acclimation period, the fish were fed the experimental diets containing varying levels of boric acid along with the control diet for a duration of 140 days. Throughout both the acclimation and feeding trials, the fish were fed twice a day until apparent satiety. Daily monitoring of water quality parameters ensured that temperatures ranged between 9 and 13 °C, and dissolved oxygen levels remained above 9 mg/l throughout the experimental period.

The feed treatments were batch-prepared in 10 kg quantities. Initially, the powdered boric acid was diluted with 500 mL of water and incorporated into the feeds through spraying and impregnation. Subsequently, the feeds were washed to lubricate them, preventing the boric acid from leaching into the water. Finally, the feed batches were air-dried in the shade and stored in covered buckets. The water lubrication method was similarly applied to the control group.

### Calculations of Growth-Related Performance

The specific growth rate (SGR, % day^−1^) is calculated as:

$$100\hspace{0.17em}\times \hspace{0.17em}(\text{ln w}1\hspace{0.17em}-\hspace{0.17em}\text{ln w}0)/\text{t},$$

where w1 and w0 are the wet weights at times t1 and t0.

The feed conversion ratio (FCR) is computed as:

$$({\text{W}}_{\text{final}}\hspace{0.17em}-{\hspace{0.17em}\text{W}}_{\text{initial}})/\text{consumed feed},$$

where W_final_ and W_initial_ are the live weights (g) of the fish on the initial day (t) and the final (T) day.

The protein efficiency ratio (PER) is calculated by dividing weight gain (g) by the protein intake (g) [25].

At the conclusion of the feeding trial, the fish were anesthetized using 300 ppm of 2-phenoxyethanol and promptly wiped with 70% ethanol. Blood samples were then collected from the vena caudalis using heparinized syringes. These samples were divided into standard lavender-top blood collection tubes containing anticoagulant (EDTA) for hematological analysis and standard red-top (SST™ II) advance serum separator tubes for serum biochemical parameter analysis. The latter samples underwent centrifugation at 13,000 × g for 10 min at 4 °C to obtain serum. Hematological parameters were analyzed immediately, while serum biochemical samples were stored at -80 °C until analysis.

Red blood cell (RBC), mean cell volume (MCV), mean cell hemoglobin (MCH), mean cell hemoglobin concentration (MCHC), hematocrit (Hct), and hemoglobin (Hb) were analyzed using a hematology auto analyzer MS4-S (Melet Schloesing Laboratories, Osny, France). To ensure the accuracy of the automated blood count device results, a manual hematological analysis was conducted on all blood samples immediately after collection into K3EDTA tubes, following the method outlined by Blaxhall and Daisley [26]. Serum alkaline phosphatase (ALP), glutamic oxaloacetic transaminase (GOT), glutamic pyruvic transaminase (GPT), glucose (GLU), albumin (ALB), cholesterol (CHOL), total protein (TPR), and globulin (GLO) levels were measured using a biochemical analyzer (MScan II, Melet Schloesing, Osny, France).

### Oxidative Stress Parameters

TAS levels were measured using commercially available kits from Relassay (Cat no:RL0017), Turkey [27]. Determination of Total Oxidant Status (TOS) levels were assessed using commercially available kits from Relassay (Cat no:RL0024), Turkey [28]. The ratio of TOS to TAS is accepted as the oxidative stress index (OSI. For calculation, the resulting unit of TAS is converted to μmol/L, and OSI value is calculated according to the following formula: OSI (arbitrary unit = TOS (μmol H2O2 equivalent/L / TAC (μmol Trolox equivalent/L [29–31]. As a product of lipid peroxidation, the level of malondialdehyde (MDA) was determined according to [32]. The measurements of erythrocyte and tissue supernatant myeloperoxidase (MPO) enzyme activity were performed according to [33]. The protein concentration of each sample was determined spectrophotometrically at 595 nm based on Bradford method [34].

### Statistics

The data obtained from each treatment group were analysed using a one-way ANOVA, followed by post hoc Tukey's honestly significant difference (HSD) tests. The statistical analyses were performed using SPSS 18.0 software (Illinois, USA). There is a notable distinction when the *p*-value is < 0.05.

## Results

The present study calculated the growth performance of a portion-size rainbow trout fed with the feeds containing boric acid at different rates for 140 days. The results are presented in Table 1. The study revealed that the inclusion of boric acid in the diet positively impacted the growth performance of rainbow trout. Moreover, the observed differences in growth performance among the experimental groups were found to be statistically significant at a significance level of *P* < 0.05. The rainbow trouts in the four experiment groups with an average initial weight of 397.28 g reached the final weights of 928.15 ± 5.73 g, 955.24 ± 8.67 g, 994.75 ± 7.46 g, and 976.80 ± 6.26 g, respectively, and the highest live weight gain (597.48 ± 7.46 g) was achieved in the 3rd group which was fed with 0.025% boric acid.

**Table 1:** Growth performance of the rainbow trout which were fed diets with various boric acid concentrations for 140 days

	**G1-Control**	**G2-0.010%**	**G3-0.025%**	**G4-0.050%**
IFW	397.28 ± 3.21	397.28 ± 3.21	397.28 ± 3.21	397.28 ± 3.21
FW	928,15 ± 5.73^d^	955,24 ± 8.67^c^	994,75 ± 7.46^a^	976,80 ± 6.26^b^
WG	530,87 ± 5.73^d^	557,96 ± 8.67^c^	597,48 ± 7.46^a^	579,52 ± 6.36^b^
FI	755,50 ± 4.55^a^	732,32 ± 3,17^b^	711,57 ± 3.42^c^	734,11 ± 2.35^b^
DFI	5,39 ± 0.03^a^	5,23 ± 0.02^b^	5,08 ± 0.02^c^	5,24 ± 0.02^b^
FCR	1,42 ± 0.02^a^	1,31 ± 0.01^b^	1,19 ± 0.01^d^	1,27 ± 0.01^c^
SGR	0,61 ± 0.01^d^	0,63 ± 0.01^c^	0,66 ± 0.01^a^	0,64 ± 0.01^b^
PER	1,63 ± 0.02^d^	1,77 ± 0.01^c^	1,95 ± 0.02^a^	1,84 ± 0.01^b^
SUR (%)	100	100	100	100

Each value indicates the average ± standard deviation

The averages which are expressed using different letters in each row are significantly different (*p* < 0.05)

*IFW* Initial fish weight, *FW* Final weight, *WG* Weight gain, *FI* Feed intake, *DFI* Daily feed intake, *FCR* Feed conversion rates, *SGR* Specific growth rate, *PER* Protein efficiency ratio, *SUR* Survival rate

In the present study, oxidative stress parameters were determined from the blood serum and muscle tissue samples of the fish, and the results are presented in Table 2. The sera were obtained from the blood samples taken from the fish, and the amounts of TAS, TOS, OSI, MPO, and MDA as a result of the study were determined in the blood sera. TAS, TOS, MPO, MDA, and MP in the muscle tissue were determined.

**Table 2:** Oxidative stress response in blood and muscle tissues of rainbow trout

	**G1-Control**	**G2-0.010%**	**G3-0.025%**	**G4-0.050%**
**Blood Oxida**ti**ve Stress Parameters**				
**TAS (mmol/L)**	1.14 ± 0.02^c^	1.72 ± 0.02^a^	1.72 ± 0.04^a^	1.40 ± 0.06^b^
**TOS (µmol/L)**	5.30 ± 0.33^c^	10.90 ± 0.23^b^	11.35 ± 0.62^b^	12.20 ± 0.82^a^
**OSI**	0.46 ± 0.30^c^	0.63 ± 0.00^b^	0.66 ± 0.40^b^	0.87 ± 0.09^a^
**MPO (U/L)**	241.25 ± 10.30^a^	55.50 ± 4.43^c^	54.00 ± 3.55^c^	67.25 ± 2.06^b^
**MDA (mmol/L)**	21.83 ± 2.07^c^	39.59 ± 4.70^b^	45.79 ± 2.94^b^	69.18 ± 5.78^a^
**Muscle Oxidative Stress Parameters**				
**TAS (µmol/mg protein)**	8.06 ± 0.68^b^	8.36 ± 0.26^b^	9.74 ± 0.58^a^	10.45 ± 0.38^a^
**TOS (µmol/mg protein)**	1.119 ± 0.02^a^	0.952 ± 0.01^b^	0.894 ± 0.01^c^	0.888 ± 0.01^c^
**MPO (Spesifik Aktivite /mg protein)**	1268 ± 31.56^a^	1230 ± 26.74^ab^	1191.90 ± 23.18^b^	885.17 ± 23.35^c^
**MDA (µmol/mg protein)**	406.97 ± 6.45^a^	234.60 ± 9.20^b^	129.53 ± 6.07^c^	77.21 ± 4.68^d^
**MP**	60.20 ± 2.21^a^	69.13 ± 2.38^b^	79.33 ± 4.00^a^	70.10 ± 1.92^b^

*TAS* Total Antioxidant Status, *TOS* Total Oxidant Status, *OSI* Oxidative Stress Index, *MPO* Myeloperoxidase, *MP* Mıcroprotein, *MDA* Malondialdehyde

Lowercase superscripts (a, b, c) indicate significant differences on the same colon within each experimental treatment group (*n* = 9, Mean ± Standard devition)

TAS is 1.14 (mmol/L) in the control group. The boric acid which was added to the diet is found to change TAS levels of rainbow trout, and it varies between 1.72 and 1.40 (mmol/L) in the treatment groups. The boric acid decreased the Total Oxidant Status (TOS) of the rainbow trout, and there is a statistical difference between the study groups and the control group (*P* < 0.05). The results were calculated as 5.30 ± 0.33, 10.90 ± 0.23, 11.35 ± 0.62, and 11.35 ± 0.62 µmol/L, for G1, G2, G3, and G4, respectively. In addition, boric acid feed caused an increase in Malondialdehyde level and a decrease in Myeloperoxidase level in the blood serum. The analyses of the muscle samples taken as a result of the research concluded that boric acid added feed caused an increase in TAS and MP levels of rainbow trout, while it caused a decrease in TOS, MPO, and MDA levels.

At the end of the feeding period, blood samples were taken from the rainbow trout which were fed with different rates of boric acid-supplemented feed for 140 days. The results of the hematology and serum biochemistry parameters are presented in Tables 3 and 4.

**Table 3:** Hematological parameters of rainbow trout which were fed diets with various boric acid concentrations for 140 days

	**G1-Control**	**G2-0.010%**	**G3-0.025%**	**G4-0.050%**
RBC (m/mm^3^)	1.77 ± 0.07^a^	1.50 ± 0.04^b^	1.31 ± 0.02^c^	1.29 ± 0.05^c^
MCV (fl)	224,47 ± 13.90^b^	335,44 ± 14.53^a^	340,42 ± 8.50^a^	343,20 ± 9.10^a^
MCH (pg)	66,81 ± 4.96^b^	119,52 ± 4.34^a^	114,11 ± 4.25^a^	113,68 ± 9.00^a^
MCHC (g/dl)	29,79 ± 1.85^b^	35,68 ± 1.92^a^	33.55 ± 1.81^a^	33,18 ± 3.24^a^
Hct (%)	39,76 ± 1.39^c^	50,32 ± 2.62^a^	44,60 ± 1.43^b^	44,38 ± 1.81^b^
Hb (g/dl)	11,83 ± 0.55^c^	17,92 ± 0.41^a^	14,95 ± 0.65^b^	14,70 ± 1.26^b^

Each value indicates the average ± standard deviation. The averages which are expressed using different letters on each row are significantly different (*p* < 0.05)

*RBC* Red blood cell, *MCV* Mean cell volume, *MCH* Mean cell haemoglobin, *MCHC* Mean cell hemoglobin concentration, *Hct* Hematocrit, *Hb* Hemoglobin

**Table 4:** Serum biochemical parameters of rainbow trout which were fed diets with various boric acide concentrations for 140 days

	**G1-Control**	**G2-0.010%**	**G3-0.025%**	**G4-0.050%**
ALP (U/l)	191.67 ± 2.52^d^	207,33 ± 3.05^c^	224.00 ± 2.64^b^	239,67 ± 4.04^a^
GOT (U/l)	433,67 ± 7.57^a^	412.00 ± 1.00^b^	398,33 ± 2.52^c^	385,67 ± 1.52^d^
GPT (U/l)	29,67 ± 1.15^a^	24,67 ± 1.15^b^	19,33 ± 0.57^c^	17.00 ± 1.00^d^
GLU (mg/dl)	89.00 ± 2.00^a^	87.00 ± 2.64^a^	76,33 ± 2.51^c^	81.00 ± 2.00^b^
ALB (g/dl)	0.38 ± 0.01^d^	0.49 ± 0.03^c^	0,57 ± 0.01^b^	0,63 ± 0.03^a^
CHO (mg/dl)	282,33 ± 2.89^a^	259,33 ± 1.53^c^	245,67 ± 2.08^d^	271,33 ± 4.04^b^
TPR (g/dl)	3,37 ± 0.06^d^	4,23 ± 0.05^b^	4,47 ± 0.05^a^	3,73 ± 0.05^c^
GLO (g/dl)	3,23 ± 0,06^d^	3,60 ± 0.10^b^	3,83 ± 0.05^a^	3,47 ± 0.06^c^

Each value indicates the average ± standard deviation. The averages which are expressed using different letters on each row are significantly different (*p* < 0.05)

*ALP* Serum alkaline phosphatase, *GOT* Glutamic oxaloacetic transaminase, *GPT* Glutamic pyruvic transaminase, *BUN* Blood urea nitrogen, *GLU* Glucose, *ALB* Albümin, *CHOL* Cholesterol, *CRE* Creatinine, *TBL* Total bilirubin, *TPR* Total protein, *GLO* Globulin, *URA* Uric acid

## Discussion

The two most common measures of responses in fish to a particular diet or component are growth and feed use. Growth is measured in the function of specific nutrient gain (e.g., protein), weight gain, or length. One goal of aquaculture is to maximize production in terms of biomass. Therefore, body weight gain is a common performance measure [35]. [12, 13] had positive results in their feeding study with boric acid but emphasized the necessity of performing feeding trials with different size groups and doses. They could not foresee what results would be obtained in long-term feeding studies [12, 13]. In the present study, lower doses of boron were used compared to previous feeding studies, and five-time feeding was applied for a relatively long time. In addition, nutritive elements are usually investigated in smaller fish. The present study used rainbow trout of portion size, and very promising results were obtained regarding growth performance.

Many active substances are tested to increase the growth performance of fish. Öz and Dikel reported that garlic increases the growth performance in their 2022 study with garlic-supplemented feed [36]. In a study conducted by Öz et al. [37], it was observed that the administration of black cumin oil results in enhanced growth performance and improved immune system functionality in nile tilapia. It was reported in another study that excess glycine and glutamate in the rainbow trout diet improves the digestibility of proteins, lipids, and most of the amino acids and fatty acids, which is positively reflected in their growth performance [38]. The second factor that indicates performance in feeding trials is feed use. It refers to the extent to which the organism's food is converted to growth, which is especially important when comparing the economic cost and potential of feeds to contaminate the culture medium [35]. The feed utilisation of the research groups was determined as 755.50 ± 4.55 g, 732.32 ± 3.17 g, 711.57 ± 3.42 g, and 734.11 ± 2.35 g. The present study revealed that the group which received the feed supplementation of 0.025% boric acid exhibited the lowest feed conversion ratio (FCR) as 1.19. Conversely, the control group demonstrated the highest FCR ratio as 1.42.

A combination of dietary cinnamon and a probiotic has been reported to improve immunity, digestive enzyme activity, and growth performance of rainbow trout under stress from stockpiles. While the highest FCR in the aforementioned study was calculated in the control group (1.53), it was reduced to 1.25 in the treatment groups [39]. Similarly in the present study, the fish which were fed with boric acid supplementation demonstrated higher final weight, weight gain, and lower food conversation ratio than the individuals which were fed non-supplemented feed, indicating that boric acid in the diet exerts a growth-promoting effect. Consistent with these research findings, some studies also report the effects of nutritional supplements on fish growth. Naderi et al. [40] reported that dietary vitamin E contributes to the growth performance of rainbow trout. Additionally, Dikel et al. found in [41] that L-carnitine addition to rainbow trout feeds affects growth performance positively.

TAS in fish indicates the overall antioxidant capacity and protection against oxidative stress in their tissues caused by free radicals and reactive oxygen species [42]. TAS assessment is crucial to evaluate the protective effects of antioxidants against oxidative stress and to understand the impact of oxidative stress on fish physiology [43]. Moreover, TAS has been used to assess the effects of dietary supplementation on some fish such as the evaluation of the effects of different dietary vitamin E levels on growth performance, non-specific immune responses, and disease resistance in parrot fish [44]. The addition of compounds or chemicals to fish diets can significantly impact Total Antioxidant Status (TAS) of fish. For instance, dietary inclusion of astaxanthin has been found to increase TAS in fish, indicating its strong antioxidant properties [45]. On the other hand, the addition of *Oregano vulgare* extract to fish diets has been shown to decrease the activity of antioxidant enzymes and total antioxidant capacity [46]. Conversely, the inclusion of betaine in plant-protein-based diets has been found to enhance the antioxidant status in fish [47]. Consequently, additives to fish diets may affect their Total Antioxidant Status (TAS) differently. While compounds such as astaxanthin can boost TAS, others such as *Oregano vulgare* extract may reduce it. The type and quantity of fish oil, along with the presence of additives such as tannic acid and betaine, also influence TAS. Additionally, the composition of the basal diet impacts the fish's antioxidant status significantly. In the present study, significant increases in TAS levels are observed in G2 and G3 (1.72 mmol/L) compared to the control group (G1), while this increase was slightly lower (1.40 mmol/L) in G4. It indicates that the feeds with boric acid can enhance antioxidant capacity up to a certain dosage. TAS levels in muscle tissue observed a slight increase in G2 and G3, with a more pronounced increase in G4 (10.45 μmol/mg protein). It suggests that the feeds which contain boric acid may improve antioxidant capacity in muscle tissue.

Total Oxidant Status (TOS) in fish refers to the measurement of the different oxidant species present in the biological samples, providing valuable insights into the oxidative status and the presence of oxidant compounds. In conjunction with Total Antioxidant Capacity (TAC), TOS has been proposed as a valuable endpoint to assess the oxidative status in fish after exposure to chemicals, reflecting the combined action of different oxidants present in the biological sample [48]. Furthermore, TOS has been utilized in various studies to assess the oxidative stress and redox balance in fish, which poses significance in understanding the impact of oxidative stress on fish physiology [49, 50]. For instance, the inclusion of *Oregano vulgare* extract in fish diets has been found to decrease the activity of antioxidant enzymes and total antioxidant capacity, thereby affecting TOS [46]. Oxidative Stress Index (OSI) in fish serves as a comprehensive indicator of the degree of oxidative stress, providing valuable insights into the balance between total oxidant status (TOS) and total antioxidant capacity (TAC). OSI is calculated as the ratio of TOS to TAC, making it a reliable measure of the oxidative status in fish. The addition of compounds or chemicals to fish diet can have a significant impact on their Oxidative Stress Index (OSI). The use of various dietary carbohydrate and lipid sources has been found to affect the oxidative status, as evidenced by changes in antioxidant enzyme activities, lipid peroxidation, and OSI in European sea bass juveniles [51]. Furthermore, the quality of dietary oils has been demonstrated to influence OSI in Atlantic cod, and various dietary oil qualities affect oxidative stress as indicated by changes in lipid peroxidation and antioxidant enzyme activities [52]. In the blood samples in the present study, the values of TOS and OSI were elevated in G2, G3, and G4 compared to G1. This increase suggests that the feeds containing boric acid may increase oxidative stress.

Myeloperoxidase (MPO) is an enzyme found in neutrophils and a marker of neutrophil activation and inflammation. It is involved in the production of hypochlorous acid, a potent antimicrobial agent. In fish, MPO activity has been detected in various tissues and is used as an indicator of neutrophil activation and degranulation [53]. Malondialdehyde (MDA) is a naturally occurring product of lipid peroxidation and commonly used as an indicator of oxidative stress and lipid peroxidation in fish. It is considered as a basic compound of cellular damage by toxins, and it is a biomarker of oxidative stress occurring in cell components [54]. Microprotein (MP) is a term used to refer to small proteins or peptides. The addition of compounds or chemicals to fish diet may have a significant impact on various biomarkers, including Myeloperoxidase (MPO), Malondialdehyde (MDA), and Microprotein (MP). For instance, the inclusion of dietary probiotic bacteria and processed yeast has been shown to influence MPO activity, which plays a role in regulating reactive oxygen species (ROS) and killing invading pathogens through the production of hypochlorous acid and strong oxidants [55]. Furthermore, the addition of plant essential oils as fish diet additives has been reported to enhance fish health and stability in feed, potentially impacting MDA and overall health status [56]. In the present study, the levels of MPO in blood samples decreased significantly in G2 and G3 but were reduced less in G4. MDA levels increased across all treatment groups, indicating an increase in lipid peroxidation. In muscle tissue, both TOS and MPO levels decreased in all treatment groups compared to G1. It indicates that feeds with boric acid may reduce oxidative stress and inflammation in muscle tissue. The levels of MDA decreased in all treatment groups compared to G1, while MP levels increased in G2 and G4 groups. It shows that feeds which contain boric acid may reduce lipid peroxidation and preserve protein structure in muscle tissue. The present study demonstrates that the feeds which are supplemented with boric acid may affect oxidative stress parameters in the blood and muscle tissues of rainbow trouts in various ways. Boric acid at certain doses can enhance antioxidant capacity and also increase oxidative stress and lipid peroxidation. These findings highlight the importance of further research on the potential benefits and risks of boric acid.

Haematology is a significant method to assess the health of fish in connection to several factors such as diseases, stress, nutrition, and alterations in environmental circumstances [57–59]. Dietary boric acid decreased the red blood cell (RBC) value of the rainbow trout. It was found to be 1.77 ± 0.07, 1.50 ± 0.04, 1.31 ± 0.0, and 1.29 ± 0.05 for the three experiment groups. In their study, Fazio et al. observed RBC values ranging between 1.55–4.55 in the blood samples from the trout farms in Italy and Turkey, and the present results are close to those obtained from the rainbow trout in Turkey [60]. According to the present research findings, boric acid has the ability to impact hematopoietic tissue, resulting in a reduction in the release of red blood cells (RBCs) into the circulatory system. Based on the findings presented by Joshp et al. [61], the experimental groups exhibited a notable reduction in red blood cell (RBC) count compared to the control group. This decrease may result from the inhibition of erythropoiesis and an elevated rate of erythrocyte breakdown in hematopoietic organs. In fish, RBCs absorb oxygen in the gills and release it into the tissues. A decrease in RBC below normal limits causes insufficient oxygen to be carried to the tissues. In the current study, although boric acid reduced the RBC value of rainbow trout compared to the control group, RBC remained within normal limits. Boric acid may adversely affect the health of fish in higher doses and long-term use.

The hematocrit (Hct) and haemoglobin (Hb) levels of the fish displayed a slight initial rise which was followed by a subsequent decline in response to the increasing concentration of boric acid. The groups that were fed boric acid-supplemented feed exhibited higher values of Mean cell volume (MCV), Mean cell haemoglobin concentration (MCHC), and Mean cell haemoglobin (MCH) in comparison to the control group. However, no difference was determined among the treatment groups. The hematological parameters of the present study are in line with the previous studies with *Oncorhynchus mykiss* and *Salmo trutta macrostigma* species [49, 50, 60].

It was determined that boric acid addition to fish feed at increasing rates caused an increase in Alkaline phosphatase (ALP) activity compared to the control group. When the boron-applied groups are compared with each other and the control group, the results are significantly different (*p* < 0.05). Fasting blood Glc measurement is important in determining the health status of living things and in the follow-up of diseases. Glc level is important in the follow-up of the health status of many vital tissues, especially the liver [62]. The main source of Glc is foods of exogenous origin. However, fish use Glc produced in gluconeogenesis and glycogenolysis metabolic pathways with hormonal control unlike other creatures since they cannot directly use dietary carbohydrates [63]. Blood Glc levels (hyper- and hypo-glycemia) are a sensitive and reliable indicator of physiological response to nutrients and/or toxic pollutants that cause environmental stress in fish [64]. As the principal energy substrate, blood glucose plays a pivotal part in the metabolic processes of fish. According to Heath [65], the analysis of blood glucose levels in fish can provide valuable insights on various aspects such as their metabolic status, rate of generation and consumption, and energy requirements.

The biochemical and pathophysiological characteristics of albumin and globulins play a crucial role in the assessment of health status, disease diagnosis, and therapy monitoring. These proteins are integral components of the total protein panel. Evaluation of protein content is used as a good diagnostic tool to determine the physiological state of cells [62]. The average values of some commonly used biochemical parameters in various healthy fish species were analysed in a review study published in 2007 which reported total protein as 3.49 ± 1.007 (0.10–7.50) g/dL, albumin as 1.23 ± 0.639 (0.10–3.20) g/dL, globulin as 2.38 ± 0.664 (0.40 -4.37) g/dL, urea as 5.33 ± 4.056 (0.00–18.00) mg/dL, cholesterol from blood lipids as 248.62 ± 146.248 (0.10–714.29) mg/dL, and triglycerides as 225.90 ± 175.995 mg/dL [66]. In the present study, boric acid which was supplemented to fish feed changed the biochemical values of blood samples. Likewise, previous studies obtained similar results [21–24, 67, 68].

## Conclusion

In the current research, different proportions of boric acid were added to portion-size rainbow trout feed, and the fish were fed for 140 days. Boric acid added to the feed at the end of the feeding period positively affected the growth performance of large rainbow trout. It did not have a negative effect on blood hematology and biochemistry parameters. These findings highlight the potential advantages of using boric acid as a supplementary substance in the diet of rainbow trout, since it has been shown to promote their growth while maintaining their haematological and biochemical well-being. Further studies are needed to determine the optimal application frequency of substances such as boric acid and to evaluate their effects on the immune system and infection with parasites or other diseases.

## Data Availability

No datasets were generated or analysed during the current study.
